# Methicillin-resistant Staphylococcus aureus Meningitis as a Complication of Facial Nerve Decompression for Vertebrobasilar Dolichoectasia

**DOI:** 10.7759/cureus.3392

**Published:** 2018-10-01

**Authors:** Yash Jobanputra, Purva Sharma, Sean J Martinez

**Affiliations:** 1 Internal Medicine, University of Miami Miller School of Medicine, Atlantis, USA; 2 Radiology, VA Medical Center, Riviera Beach, USA

**Keywords:** vertebrobasilar dolichoectasia, mrsa meningitis, hemi-facial spasms

## Abstract

We present a case of a 55-year-old lady with intermittent twitching of the left side of her face, involving her left eyelid and the angle of the mouth, ongoing for two years. She failed multiple trials of botulinum toxin injections as well as oral anti-spasmodic medications. The patient was diagnosed with an ectatic left vertebral artery, causing a compression of cranial nerve VII on the same side on magnetic resonance imaging (MRI) of the brain. She underwent neurosurgery with a microvascular decompression of the ectatic artery with a resolution of hemifacial spasms. However, her postoperative course was complicated by headaches and low-grade fevers. She also had leukocytosis on a laboratory evaluation. The postoperative computed tomography (CT) scan of her head was normal. The patient had a lumbar puncture done, which showed an elevated white cell count in cerebrospinal fluid (CSF) analysis and the CSF culture was positive for methicillin-resistant Staphylococcus aureus (MRSA) bacteria. She was diagnosed with MRSA meningitis as a postoperative complication following microvascular decompression. The patient had a revision surgery of the decompression site, including wound debridement and did well postoperatively.

## Introduction

Vertebrobasilar dolichoectasia (VBD) is characterized by the elongation and dilatation of the vertebrobasilar arteries. The prevalence of VBD is between 0.2 to 4.4% [1-2]. VBD can present as ischemic stroke, which is one of the most common manifestations [3]. It also presents as a symptomatic compression of the brainstem or cranial nerves. One such presentation is hemifacial spasms (HFS). HFS due to the direct compression of the facial nerve by a dolichoectatic vertebrobasilar artery is rare [4-5]. A hemifacial spasm is described as a movement disorder characterized by involuntary paroxysmal facial movements. Typical HFS usually involves the orbicularis oculi and then gradually spreads to the other facial muscles over time. The treatment of hemifacial spasms in cases of VBD involves medical management with botulinum toxin injections or by surgical microvascular decompression of the facial nerve. We present one such case of HFS due to VBD managed with microvascular decompression and resulting in a complication of methicillin-resistant Staphylococcus aureus (MRSA) meningitis and wound infection.

## Case presentation

A 55-year-old female veteran presented with complaints of intermittent twitching of her left face. The twitching has been ongoing for the last two years and had been progressively worsening over a period of time. The twitching involved the eyelid and the angle of the mouth on the left side. She was initially treated with antispasmodic medications, which failed to provide relief. She also failed trials of botulinum toxin injections locally to treat the spasms.

Her past medical history was significant for hypertension and hyperlipidemia. She was on three antihypertensives for blood pressure control, which included amlodipine, metoprolol, and chlorthalidone. Her only other medication was a low-dose aspirin daily. She also smoked cigarettes, approximately one pack per day for more than 20 years. She is a veteran and served in the airforce in the post-Vietnam era.

**Figure 1:**
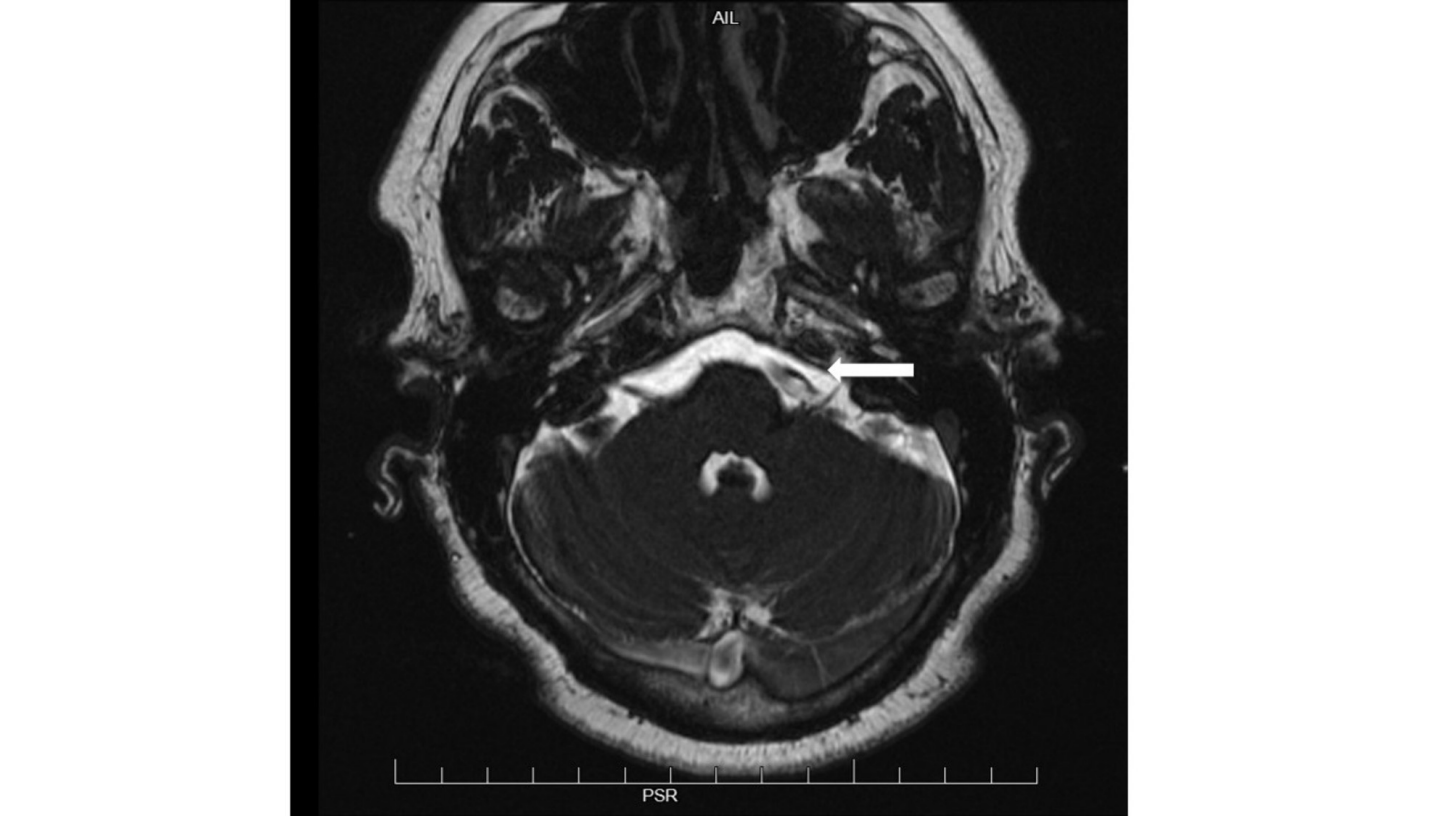
MRI of brain showing an ectatic left vertebral artery (arrow)

On arrival to the emergency department, the patient was afebrile, with heart rate (HR) 80 beats/minute, blood pressure (BP) 146/84 mmHg, and she was saturating 95% on room air. Her physical examination was unremarkable apart from the occasional twitching noticed on the left side of her face. The laboratory evaluation was also unremarkable. Magnetic resonance imaging (MRI) of the brain with and without intravenous contrast showed an ectatic left vertebral artery compressing cranial nerve seven as it exited from the brainstem. (Figure 1) The patient was referred to neurosurgery. She underwent a left-sided craniotomy and microvascular decompression of the dolichoectatic vertebrobasilar junction using Teflon paddies. The patient did well postoperatively, with an almost immediate resolution of the hemifacial spasms.

However, on Day 3 post-procedure, she started complaining of severe headaches. The headaches were associated with nausea, vomiting, left-sided earache, and low-grade subjective fevers. She was afebrile and hypertensive with a blood pressure of 170/100 mmHg. On physical examination, she appeared to be in distress and sutures were present in the left posterior auricular area. The rest of the examination was within normal limits. Her laboratory evaluation was remarkable for leukocytosis of 22x103/uL with 82.5% neutrophils. In this clinical setting, a postoperative infection was suspected and the patient was started on broad-spectrum antibiotic coverage with intravenous (IV) vancomycin and cefepime. A computed tomography (CT) scan of the head showed no acute intracranial pathology. A lumbar puncture was done and a cerebrospinal fluid (CSF) analysis was significant for an elevated white count of 3077 with 69% neutrophils, CSF glucose was low (12mg/dL), and protein was elevated (200mg/dL). The culture from the CSF grew MRSA and the patient was diagnosed with MRSA meningitis as a post-operative complication. On post-op Day 7, sutures from the surgical site were removed and about 100 ml of purulent fluid was drained. The culture from the wound was also MRSA positive. The wound was debrided again and the patient was continued on IV vancomycin for a total of six weeks. She responded appropriately to the antibiotic and did well with a resolution of her symptoms.

## Discussion

Vertebrobasilar dolichoectasia is a rare disease characterized by significant expansion, elongation, and tortuosity of the vertebrobasilar arteries. Although there is no current data on the exact incidence of VBD in the general population, angiography and autopsy results suggest that the overall incidence is less than 0.05% [3]. The etiology of VBD is unclear, however, there are certain risk factors associated with the disease. Increasing age, hypertension, and smoking are some of the known risk factors. Of note, our patient had at least two risk factors, including hypertension controlled on three medications, as well as a prolonged history of tobacco use.

VBD can cause an ischemic stroke and it is one of the most common causes of VBD-related death. VBD commonly causes mass effect, causing compressive side-effects to the brainstem as well as the cranial nerves. Nearly all cranial nerve damage symptoms can be associated with VBD [6]. Two of the most common ones are trigeminal neuralgia and hemifacial spasms.

Hemifacial spasms are involuntary painless spasms, involving the orbicularis oculi and orbicularis oris muscles of the face. It is usually caused by the direct or indirect compression of the seventh cranial nerve at the root exit zone in the brain stem. The compression is mostly caused by vascular loops, however, it could sometimes be caused by a tumor, cyst, or an aneurysm of one of the arteries in the posterior cerebral circulation [7]. All patients with HFS must undergo magnetic resonance imaging (MRI) studies including magnetic resonance angiography (MRA) with close attention to the seventh cranial nerve to evaluate for a secondary cause of the disease such as VBD.

The treatment of HFS includes anti-spasmodic medications, as well as botulinum toxin injections in the muscles locally, which is highly effective and has minimum side-effects. However, sometimes, the symptoms of VBD could be refractory to medical therapy and need surgical intervention. Radiofrequency ablation, gamma knife, and percutaneous balloon compression of the affected nerve are some of the effective methods of treatment [8]. Because all compression symptoms in VBD result from the pulsating nerve root compression, the most effective treatment is microvascular decompression [5].

Microsurgical vascular decompression is the curative treatment and is effective in 70%-95% of patients [7]. However, it is amenable to complications such as hearing loss, severe facial paralysis, infections of the central nervous system (CNS), and death [5,9]. El-Ghangour et al. studied 10 patients with VBD who were treated by microvascular decompression [10]. All the 10 patients had trigeminal neuralgia and four of them had associated HFS. Three out of the four (75%) patients with HFS achieved a complete resolution of their symptoms. The only complication seen in one patient was a mild facial weakness. No recurrence was found in an average of 7.8 years of follow-up. In addition, compared to the reposition and fixation surgery, the microvascular decompression operation was relatively easy with low technical requirements and fewer complications.

Meningitis is a known complication of microvascular decompression. In this case, we report meningitis as a potential complication in patients with VBD who are treated with microvascular decompression. Our patient grew MRSA bacteria in the CSF culture as well as from the surgical site, indicating that it is an important postoperative complication following a neurosurgical procedure.

MRSA remains one of the most important causes of antimicrobial-resistant healthcare-associated infection (HCAI) worldwide and is an important cause of bloodstream infection (BSI) and other invasive infections [11-13]. A retrospective review of invasive MRSA infections in neurosurgical patients was conducted at a large tertiary neurosurgical center in Europe [14]. Forty-four cases of invasive MRSA infection were identified in neurosurgical patients over the study period. Of these, meningitis was found in 18% of the patients and was the second most common infection after bloodstream infection (BSI). Thus, MRSA meningitis is a known complication of neurosurgical procedures, which can also result from microvascular decompression surgery.

## Conclusions

Vertebrobasilar dolichoectasia is the dilatation and elongation of the vertebrobasilar arteries. It can result in the compression of one or more cranial nerves. Compression of the facial nerve (CN VII) can result in hemifacial spasms. Microvascular decompression surgery is an effective treatment modality for the treatment of hemifacial spasms. However, patients can have complications such as hearing loss, facial paralysis, and infections. Meningitis can be one of the potential complications of decompressive surgery and should be considered among the differential diagnosis in these patients.
